# CGRP Administration Into the Cerebellum Evokes Light Aversion, Tactile Hypersensitivity, and Nociceptive Squint in Mice

**DOI:** 10.3389/fpain.2022.861598

**Published:** 2022-04-25

**Authors:** Mengya Wang, Thomas L. Duong, Brandon J. Rea, Jayme S. Waite, Michael W. Huebner, Harold C. Flinn, Andrew F. Russo, Levi P. Sowers

**Affiliations:** ^1^Department of Neuroscience and Pharmacology, University of Iowa, Iowa City, IA, United States; ^2^Department of Molecular Physiology and Biophysics, University of Iowa, Iowa City, IA, United States; ^3^Center for the Prevention and Treatment of Visual Loss, Veterans Administration Health Center, Iowa City, IA, United States; ^4^Department of Neurology, University of Iowa, Iowa City, IA, United States

**Keywords:** migraine, CGRP, cerebellum, light aversion, anxiety, pain

## Abstract

The neuropeptide calcitonin gene-related peptide (CGRP) is a major player in migraine pathophysiology. Previous preclinical studies demonstrated that intracerebroventricular administration of CGRP caused migraine-like behaviors in mice, but the sites of action in the brain remain unidentified. The cerebellum has the most CGRP binding sites in the central nervous system and is increasingly recognized as both a sensory and motor integration center. The objective of this study was to test whether the cerebellum, particularly the medial cerebellar nuclei (MN), might be a site of CGRP action. In this study, CGRP was directly injected into the right MN of C57BL/6J mice via a cannula. A battery of tests was done to assess preclinical behaviors that are surrogates of migraine-like symptoms. CGRP caused light aversion measured as decreased time in the light zone even with dim light. The mice also spent more time resting in the dark zone, but not the light, along with decreased rearing and transitions between zones. These behaviors were similar for both sexes. Moreover, significant responses to CGRP were seen in the open field assay, von Frey test, and automated squint assay, indicating anxiety, tactile hypersensitivity, and spontaneous pain, respectively. Interestingly, CGRP injection caused significant anxiety and spontaneous pain responses only in female mice, and a more robust tactile hypersensitivity in female mice. No detectable effect of CGRP on gait was observed in either sex. These results suggest that CGRP injection in the MN causes light aversion accompanied by increased anxiety, tactile hypersensitivity, and spontaneous pain. A caveat is that we cannot exclude contributions from other cerebellar regions in addition to the MN due to diffusion of the injected peptide. These results reveal the cerebellum as a new site of CGRP actions that may contribute to migraine-like hypersensitivity.

## Introduction

Migraine is a neurological disease that affects about 15% of the population (1) and is the second leading cause of disability globally (2). It is characterized by moderate or severe headaches that are accompanied by sensory abnormalities, such as photophobia and allodynia (3). Prevalence in women is about twice as high as in men (1). Despite its high prevalence and large burden to society, the mechanism underlying migraine have yet to be fully elucidated. Over the last few decades, calcitonin gene-related peptide (CGRP) has moved to the forefront in migraine pathophysiology. CGRP levels are elevated in both the ictal and interictal phases in human studies (4–6) and infusion of CGRP induced migraine-like headaches in ~66% of migraine patients (6, 7). Most recently, CGRP-based drugs have been shown to effectively alleviate migraine symptoms in about 50% of patients (8, 9). However, despite the significant advancement of CGRP-based drugs as migraine therapeutics, there is uncertainty regarding the mechanisms by which CGRP induces migraine, especially as to where CGRP is acting.

The human studies measuring CGRP levels (4, 5) and induction of migraine-like headaches by intravenous CGRP injections (7) suggest a peripheral site of action for CGRP in migraine. In addition, the antibodies targeted against CGRP or CGRP receptors have limited ability to cross the blood-brain barrier (10). However, previous animal studies demonstrated that peripheral (intraperitoneal, i.p.) (11) and central (intracerebroventricular, i.c.v.) (12) injection of CGRP induced similar light-aversive behaviors in wild-type mice. Both behaviors could be attenuated by triptan migraine drugs (11, 12). Moreover, transgenic mice overexpressing a CGRP receptor subunit in the nervous system displayed light aversion in response to dim light after i.c.v. CGRP injection (11, 13, 14), while bright light was required to induce light aversion in wild-type mice after i.c.v. CGRP injection (12). Those data suggest that increased sensitivity to CGRP in the nervous system can cause migraine-like light-aversive behavior in mice. Finally, it was found that CGRP injection into the posterior thalamic nuclei, an integration center for light and pain signals, was sufficient to induce light aversion in wild-type C57BL/6J mice, even in dim light (15). Together, these data suggest that CGRP can work in the central nervous system to induce migraine-like photophobic behavior in mice.

Similar to the thalamus, the cerebellum integrates multiple sensory signals and motor events (16, 17). While the cerebellum was originally recognized for its role in motor control (18), there is mounting evidence that it also plays important roles in perceptual (19), emotional (20), and cognitive functions (21, 22). In particular, it is now appreciated that the cerebellum participates in sensory, emotional, cognitive aspects of pain, and motor control in response to pain (23). Three lines of evidence support the link between the cerebellum and migraine pathogenesis. First, changes in cerebellar activation, structure, and functional connectivity are present in episodic, chronic, and familial hemiplegic migraine patients (24). When responding to trigeminal stimuli, cerebellar activity and functional connectivity with the thalamus and cortical areas were changed (25), suggesting the cerebellum is involved in processing sensory information from the trigeminal system. Strikingly, migraine patients exhibited decreased cerebellar activation in response to trigeminal nociceptive stimuli after treatment with erenumab, a CGRP receptor antibody (26). Second, migraine patients display cerebellar symptoms, e.g., dizziness, vertigo (27), body sway (28), as well as increased body sway accompanied by increased light intensity (29). Third, the cerebellum communicates directly to migraine-related regions, such as the spinal trigeminal nucleus (30–32) and the thalamus (33) via direct neural circuits. These data hint to the importance of the cerebellum in migraine pathophysiology.

Curiously, the cerebellum has the highest binding density to CGRP receptor PET ligands in human and rhesus brains (34, 35). The canonical CGRP receptor subunits, receptor activity-modifying protein 1 (RAMP1) and calcitonin receptor-like receptor (CLR), are localized in the human, rhesus and rat cerebellar cortex (36–38) and in the medial cerebellar nuclei (MN, also known as fastigial nuclei in humans) of rats (37). CGRP is also distributed in the cerebellar cortex (36–38) and the MN (37). In addition, as one of the three deep cerebellar nuclei, the MN receives sensory information via vestibular nuclei (39) and projects to migraine-related brain regions including the thalamus (40). The MN can also be activated by noxious thermal stimuli (23). Moreover, injection of an excitatory amino acid into the MN decreased pain-related responses to visceral stimuli (41, 42). The same amino acid stimulation increased dorsal column nuclei activity in response to non-noxious somatic stimuli (43). These findings suggest that the MN, specifically CGRP receptors in the MN, may be associated with migraine pathophysiology. Thus, we hypothesized that CGRP injection into the MN might induce migraine-like behaviors in mice.

To address the role of cerebellar CGRP in migraine-like behaviors, we injected CGRP into the cerebellum centered on the MN and performed a battery of tests to assess preclinical behaviors that are surrogates of migraine-like symptoms. The results demonstrated that CGRP infusion into the MN induced light aversion, anxiety, tactile hypersensitivity, and nociceptive squinting behaviors in mice.

## Materials and Methods

### Animals

Wild-type C57BL/6J mice were obtained from Jackson Labs (Bar Harbor, ME and Sacramento, CA) at 8–12 weeks of age and were housed in groups of 2–5 per cage before surgery. A total of 55 C57BL/6J mice (28 females; 27 males) were used for this study. Female mice had an average starting body weight of 18–22 g and males were 20–25 g. Mice with cannulas were housed individually unless otherwise indicated to prevent mice from losing cannulas. All animals were housed on a 12-h light cycle with access to water and food *ad libitum*. Animal procedures were approved by the Iowa City Veterans Administration and University of Iowa Animal Care and Use Committees and performed in accordance with the standards set by the National Institutes of Health.

### Surgery

Cannulas were hand constructed from stainless steel hypodermic tubing (New England Small Tube Corporation; Supplementary Figure 1). An 8-mm guide cannula was made from a 23-gauge needle (BD PrecisionGlide™) with the ventral portion covered by a ~7-mm, 19-gauge tubing (Supplementary Figure 1A). The ~7-mm, 19-gauge tubing is ~2 mm higher than the 23-gauge needle to shield the junction between the guide's top and the dummy or injection cannula after their insertion (Supplementary Figure 1A). The dummy cannula, used to seal and keep the guide cannula free of clogs, was made by crimping a short segment of ~5-mm, 23-gauge tubing to a ~14 mm piece of 30-gauge tubing (Supplementary Figure 1B). The bottom of the 30-gauge tubing was cut to ensure that the 30-gauge segment below the ~5-mm, 23-gauge segment is 9 mm. The injection cannula was made by adhering a short segment of ~5-mm, 23-gauge tubing ~5 mm below the top of a ~20-mm piece of 30-gauge tubing with adhesive (Pacer Technology) and dental cement (Stoelting) (Supplementary Figure 1C). The bottom of the 30-gauge tubing was cut to ensure that the 30-gauge segment below the ~5-mm, 23-gauge segment is 10 mm. In this manner, the dummy cannula extended 1 mm beyond the end of the guide cannula tip when it was inserted into the guide cannula, while the injection cannula protruded 2 mm from the base of the guide cannula (Supplementary Figure 1D).

Stereotaxic implantation of a guide cannula into the MN of the right cerebellum was performed under isoflurane anesthesia (induction 5%, maintenance 1.5–2%). The coordinates for the right MN are: anterior/posterior (AP), −6.5 mm posterior to bregma; medial/lateral (ML), −0.85 mm lateral to the midline; and dorsal/ventral (DV), −2.7 mm ventral to the pial surface according to the Allen Brain Reference Atlas. Guide cannulas were implanted 2 mm above the MN (AP: −6.5 mm; ML; −0.85 mm; DV: −0.7 mm). The implants were secured with bone anchor screws (Stoelting), adhesive, and dental cement. Dummy cannulas were inserted into guide cannulas when no injection was conducted. After surgery, mice were housed individually to reduce the loss of the guide or dummy cannulas. Mice were given ~10 days to recover from the surgery before testing unless otherwise indicated.

### Drug Administration

Rat α-CGRP (Sigma-Aldrich) was diluted in 1X phosphate-buffered saline (PBS; HyClone^TM^). Mice were given either rat α-CGRP (1 μg, 5 μg/μl) or 1X PBS (200 nl) as the vehicle through injection cannulas under anesthetized status (isoflurane: induction 5%, maintenance 1.5%−2%) unless otherwise indicated (details in Von Frey test). Specifically, the dummy cannula was removed, and an injection cannula (2 mm extension from the base of the guide cannula) was inserted into the guide cannula. The injection cannula was connected to a 10 μl syringe (Hamilton) and an injection pump (Cole-Parmer Instrument Co.) via polyethylene tubing (BD Intramedic™, PE10). The injection rate was 100 nl/min for 2 min. After completing an infusion, the injection cannula was left in position for an additional 5–7 min before being withdrawn. Next, mice were returned to their home cages (individual housing) to recover for 60 min before testing unless otherwise indicated (details in Von Frey Test). The 60-min recovering period was chosen to minimize anesthesia effects (11, 12).

### Behavioral Tests

#### Light/Dark Assay

The testing chamber was a transparent, seamless open field chamber divided into two zones of equal size by a black infrared-transparent dark insert (Med Associates). The mouse activity was collected with infrared beam tracking and Activity Monitor software (Med Associates), as previously described (12, 44). Mice were tested without pre-exposure to the chamber using dim light (55 lux) after PBS or CGRP administration as described above. 1 h post-injection, mice were placed in the light zone of the light/dark chamber and data were collected for 30 min and analyzed in sequential 5 min intervals.

Motility outcomes were collected during the light/dark assay, as described previously (12, 44). Briefly, resting time was measured as the percentage of time animals did not break any new beams in each zone over the time spent in the same zone. Vertical beam breaks, an assessment of rearing behavior, was determined as the number of mice breaking the beam at 7.3-cm height in each zone, which was then normalized to the time spent in the same zone.

#### Open Field Assay

This assay is to measure anxiety-like behavior. The apparatus was the same as in the light/dark assay with the absence of the dark insert, as described previously (12, 44). Mice were placed in the middle of the open field chamber with the light intensity at 55 lux 1 h after PBS or CGRP infusion. The periphery was defined as 3.97 cm from the border with the remaining area of 19.05 × 19.05 cm as the center. The time in the center was calculated as the percentage of time spent in the center over the total time in the chamber.

#### Von Frey Test

The test is to evaluate the mechanical nociceptive threshold. For baseline experiments, mice were habituated to the room for 1 h before acclimating to an acrylic chamber (10.80 x 6.99 x 14.61 cm in W x D x H) for 1 h. The acrylic chamber was placed over a grid support (Bioseb, France). On the treatment day, investigators gently restrained the mouse and replaced the dummy cannula with an injection cannula. Then CGRP or PBS was infused via injection cannulas to the MN of the conscious and free-moving mice. Anesthesia (isoflurane) was not used since it induced a noticeable increase in the right hind paw withdrawal sensitivity in our pilot test. The reason is unclear, but one study reported that different doses of isoflurane had opposite effects on pain withdrawal sensitivity in response to thermal stimuli (45). Considering that the isoflurane effect might mask the CGRP effect, we decided to inject mice without anesthesia in the von Frey test. After injection, mice were allowed to rest in their home cages for 30 min and then placed in the acrylic chamber for another 30 min before applying von Frey filaments to their hind paws. Right and left hind paws were tested at the same time after treatment.

The investigator who applied filaments was blinded to the treatments and used the up-and-down method as previously described (46, 47). Briefly, filaments were applied for 5 s to the skin of the mouse plantar surface, with D (0.07 g) as the starting filament. A withdrawal response was considered when mice withdrew, shook, or licked the tested hind paws. The withdrawal threshold at which 50% of mice withdrew their hind paws was determined based on an established equation (46, 47). However, the threshold data produced in this method are not continuous and cannot be analyzed using parametric statistics. Thus, in order to obtain normal distribution, the 50% thresholds (g) were transformed into log format for data analysis and figure plotting.

#### Automated Squint Assay

This assay is to evaluate spontaneous pain by measuring the right-eye pixel areas recorded by a camera. Mice were acclimated to a customized collar restraint to reduce stress induced by restraint as well as struggle or head movement as described previously (48). C57BL/6J mice underwent acclimation for 20 min per session for three sessions. On the test day, after habituation to the room for 1 h, the mouse was placed in the restraint, and squint was recorded for 5 min under room light as the baseline. Then CGRP or PBS was infused into the MN via an injection cannula. The mouse was returned to the home cage to rest for 1 h, followed by another restraint and squint recording for 5 min under room light as the treatment recording. Pixel area measurement for the right eye palpebral fissure was derived every 0.1 s (10 frames per second) in the recordings using trained facial detection software (FaceX, LLC, Iowa City, IA) with the resulting values compiled with a custom MATLAB script. Individual frames containing a tracking error rate of >15% were excluded.

#### Gait Dynamic Assay

Gait dynamics were measured using the DigiGait imaging system (Mouse Specifics Inc, Boston, MA, USA). The system consists of a transparent chamber (17.14 x 5.08 x 15.24 cm in W x D x H), a transparent plastic treadmill belt, an under-mounted digital camera, a light over the chamber for camera capturing videos (~7,200 lux), software to record videos (DigiGait Imager), and an image analysis software (DigiGait Analysis).

Mice were habituated to the room for 1 h prior to any running. Mice first were placed in the chamber of the DigiGait apparatus for 1 min to allow them to explore the chamber. The belt was then turned on and mice were run at 16 cm/s, an optimal speed predetermined in C57BL/6J mice. Images of the paws were ventrally captured during the run. Each mouse ran until roughly 3–5 s of continuous gait was observed, a range sufficient to acquire adequate quantification of gait parameters. Mice underwent recordings before PBS or CGRP injection as the baseline. After injection, mice recovered in the home cages for 1 h prior to another recording. A minimum of a one-hour interval was allotted between baseline and treatment trials to allow mice to recover from the previous running.

The mouse paw prints were analyzed by DigiGait Analysis to identify stride length and frequency. A complete stride was defined as the portion of foot strike to subsequent foot strike on the treadmill belt of the same foot.

### Histology

After finishing all the behavioral tests, the injection sites were identified by the injection cannula tip, or by infusing Evans blue dye (200 nl, 1% dye, diluted in 1X PBS), or red retrograde beads (200 nl, Red Retrobeads^TM^, LumaFluor, Inc.) to confirm targeting accuracy. Fluorescein-15-CGRP (1 μg; 200 nl mixed in 1X PBS) was injected into 4 mice to determine how far CGRP could spread from the MN. 1 h post-injection, mice were deeply anesthetized with ketamine/xylazine (87.5 mg/kg/12.5 mg/kg, i.p.) and were perfused transcardially with 1X PBS and subsequently with 4% paraformaldehyde. Brains were removed and post-fixed in 4% paraformaldehyde at 4°C overnight, followed by soaking in 10, 20, 30% sucrose per 24 h in order. Brains were embedded in a tissue-freezing medium and stored at −80 °C until use. 100 μm coronal slices were collected from mouse brains injected with Evans blue. 40 μm coronal slices were collected from brains injected with red beads or fluorescein-15-CGRP. Slices from brains injected with fluorescein-15-CGRP were counterstained by incubation with TO-PRO-3 iodide. Slices were mounted onto Superfrost Plus slides (Fisher Scientific) using antifade mountant (VECTASHIELD). Images were captured using a scanning microscope (Olympus, VS120). Imaging of brains injected with Evans blue or red beads was performed using a light microscope (Olympus, CKX41) equipped with an Infinity 1 camera and processed using the INFINITY ANALYZE software (Lumenera Corporation).

### Experimental Design

To reduce the number of animals used in this study, the cannula system was used to allow the same mouse to undergo different assays. The first cohort was tested in the light/dark assay, open field assay, von Frey test, and automated squint assay. Because of the COVID-19 pandemic, the second cohort which had been exposed to the light/dark assay, open field assay and von Frey test were euthanized to minimize the burden in the animal facility. To repeat experiments in the automated squint assay, a third cohort was included. Unlike the previous two cohorts, the third cohort was first housed in groups after surgery. However, due to the high rate of dummy cannula loss in group housing conditions for about 1 week, mice were then housed individually instead, consistent with earlier cohorts. Von Frey test, gait dynamic and automated squint assays were performed in this cohort. Data from all cohorts were pooled for the final analysis.

The order of light/dark assay and open field assay was switched in the two cohorts to avoid an order bias. All the mice received the same treatment in the light/dark and open field assays to ensure the consistency. The same treatment in the light/dark assay was also given in the squint and gait dynamic assays. One cohort received crossover treatment in the automated squint assay. To ensure the withdrawal threshold in the von Frey test was comparable in control and experimental groups, mice were divided into two groups using a randomization protocol based on the baseline threshold. Two cohorts of mice received 5–6 injections of CGRP or vehicle over 2–3 months. The interval between CGRP injections ranged from 1–3 weeks. A third cohort of mice received 3 CGRP injections over 5 weeks, then had to be euthanized due to COVID-19 restrictions. The light/dark or open field assays were performed first, followed by the von Frey test and the gait dynamic assay. The automated squint assay was performed last. All behavioral experiments were performed between 7:00 a.m. and 6:00 p.m., and mice were habituated to the room for 1 h before experiments.

### Statistical Analysis

A power analysis was performed prior to experiments for sample size estimation based on previous studies from the lab and a *post-hoc* power analysis was performed to estimate the number of additional male mice needed to reach significance using ClinCalc.com. In the power analysis, an alpha of 0.05 and a power of 0.80 was used. The analysis determined that 10 mice in each group were needed. Data were analyzed using GraphPad Prism 9 and are reported in Supplementary Table 1. Significance was set at *P* < 0.05. Error bars represent ± SEM. A two-way repeated measure ANOVA was performed when data were plotted as a function of time (factor: treatment and time) for the data from the light/dark and open field assays. When the interaction was significant, Šídák's multiple comparisons test was used as the *post-hoc* analysis. An unpaired *t*-test was performed for bar graphs with scatter points to compare the effect of each treatment.

A two-way repeated measure ANOVA was performed when data were plotted as scatter plots for the von Frey, squint, and gait dynamic assays (factor: treatment and condition). When the interaction or the condition was significant, a *post-hoc* paired *t*-test was performed to compare between baseline and treatment. It should be noted that the test was not corrected for multiple comparisons. When analyzing sex differences in these behavioral outputs, absolute values in CGRP group (time in light and time in center) or changes in CGRP group by extracting respective baseline from the treatment measurements (50% thresholds (g) and mean pixel area) in each sex were compared using an unpaired *t*-test. Pearson correlation was used to evaluate the relationship between two parameters. The power of a two-way repeated measure ANOVA was calculated using SPSS statistics 28 software.

A total of 3 mice died during the surgical procedure and one mouse lost the guide cannula before running any behavioral test. Two mice from the light/dark assay and one mouse from the open field assay were excluded due to chamber recording problems. In the von Frey test, 4 total mice were excluded: 3 mice due to the blockage of injection cannulas and one mouse due to the loss of the guide cannula. In the gait dynamic assay, 2 mice were excluded due to a video recording problem. In the automated squint assay, 2 mice were excluded due to the loss of the guide cannulas, 3 mice due to the blockage of injection cannulas, and 5 mice due to the poor habituation in the restraint or poor eye recognition by the software. Mouse numbers used for each experiment are reported in the figure legends.

## Results

### Injection of CGRP Into the MN Induced Light-Aversive Behavior and Reduced Motility Under Dim Light

**Figure 1:**
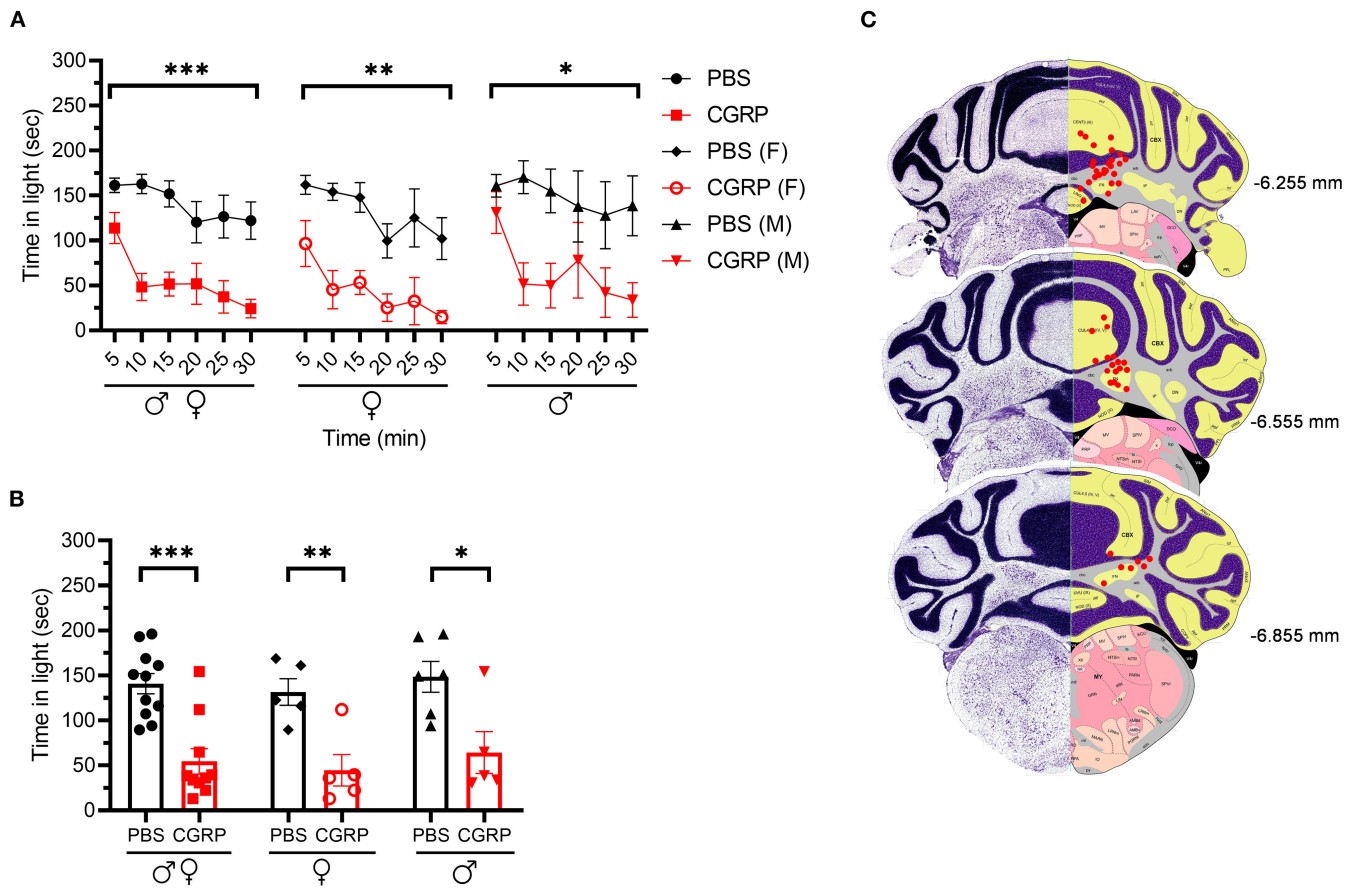
Injection of CGRP into the MN induced light-aversive behavior under dim light. **(A)** Time in light every 5-min block during 30-min light/dark assay at 55 lux following injection of PBS (*n* = 11; F: *n* = 5; M; *n* = 6) or CGRP (1 μg /200 nl; *n* = 10; F: *n* = 5; M: *n* = 5) into the right MN of C57BL/6J mice via cannulas. Time in light for all mice (left), female mice (middle), and male mice (right). Data are from two independent experiments. All mice in A are further analyzed in B. **(B)** Mean time in light per 5-min block for individual mice. **(C)** Schematic of positions of injection cannula tips superimposed on Allen Mouse Brain Atlas coronal images. Numbers indicate the distance from bregma in the anteroposterior plane. Data are the mean ± SEM. Statistics are described in Supplementary Table 1. *p ≤ 0.05, **p ≤ 0.01, ***p ≤ 0.001.

We injected CGRP into the MN of the right cerebellum via permanently placed cannulas and exposed the mice to the light/dark assay in dim light (55 lux) 1 h post-injection. Light aversion was expressed as both a function of time over the 30-min testing period (Figure 1A) and the average time in light for individual mice per 5 min interval (Figure 1B). Regardless of sex, mice injected with CGRP spent less time in the light than those injected with PBS during the 30-min testing time (Figure 1A, left). On average, the PBS-treated mice spent 141 s in the light per 5-min interval, and CGRP-treated mice spent 55 s (Figure 1B, left). When data were separated by sex, both male and female mice spent significantly less time in light after CGRP injection than those with PBS injection (Figures 1A,B, middle and right). There was no sex difference in time in light after CGRP treatment (Supplementary Table 1). For all mice, confirmation of the targeting site was performed. The injection sites for mice in all behavioral tests are shown in Figure 1C. Among 21 mice that experienced the light/dark assay, injection sites for 6 mice were not in or near the MN, primarily in the cerebellar cortex. However, these 6 off-target mice did not display significant differences in time in light from the on-target mice (data not shown). However, it should be noted that it is underpowered for off-target mice. Together, these data demonstrate that CGRP injection into the MN induces light-aversive behavior in both male and female mice.

**Figure 2:**
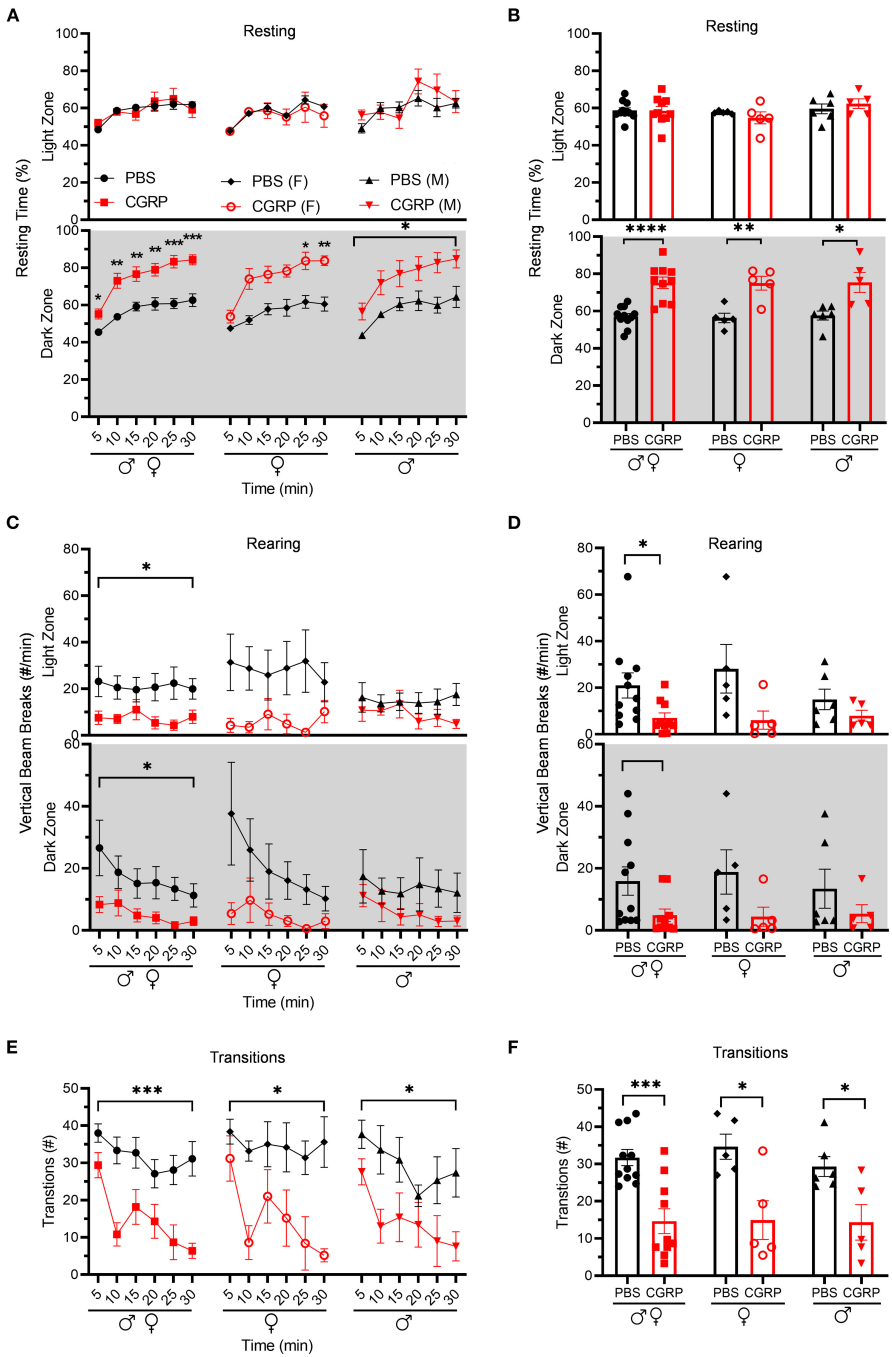
Injection of CGRP into the MN reduced motility. Motility data were collected at the same time as light aversion data from the same mice shown in Figure 1. Mice were given PBS (*n* = 11; F: *n* = 5; M: *n* = 6) or CGRP (1 μg/200 nl; *n* = 10; F: *n* = 5; M: *n* = 5) into the right MN of C57BL/6J mice via cannulas. Data are from two independent experiments. **(A)** Percentage of time spent resting in the light (upper panel) and dark (lower panel) zones every 5-min block during 30-min light/dark assay for all mice (left), female mice (middle), and male mice (right). All mice in A are further analyzed in B. **(B)** Mean percentage of time in light (upper panel) and dark (lower panel) zones per 5-min block for individual mice from A. **(C)** Number of vertical beam breaks per min in light (upper panel) and dark (lower panel) zones every 5-min block during 30-min light/dark assay for all mice (left), female mice (middle), and male mice (right). All mice in C are further analyzed in D. **(D)** Mean number of vertical beam breaks in light (upper panel) and dark (lower panel) zones per 5-min block for individual mice from C. **(E)** Number of transitions between light and dark zones every 5-min block during 30-min light/dark assay for all mice (left), female mice (middle), and male mice (right). All mice in E are further analyzed in F. **(F)** Mean number of transitions between light and dark zones per 5-min block for individual mice from E. Data are the mean ± SEM. Statistics are described in Supplementary Table 1. *p ≤ 0.05, **p ≤ 0.01, ***p ≤ 0.001, ****p ≤ 0.0001.

Resting behavior was evaluated in the same light/dark assay. No difference was observed in the percent resting time in the light zone between CGRP- and PBS-injected mice (Figures 2A,B, upper panel). In contrast, in the dark zone, CGRP-injected mice spent more time resting than PBS-injected mice across both sexes (Figures 2A,B lower panel). In addition, CGRP-injected mice had significantly fewer rearings (vertical beams breaks) in both the light and dark zones across sexes (Figures 2C,D). While there was a trend, the decreased rearing did not reach statistical significance in the male or female groups after CGRP injection, likely due to the variability and small sample size in each sex (Figures 2C,D). Finally, transitions between dark and light zones were significantly decreased by CGRP for both sexes (Figures 2E,F).

**Figure 3:**
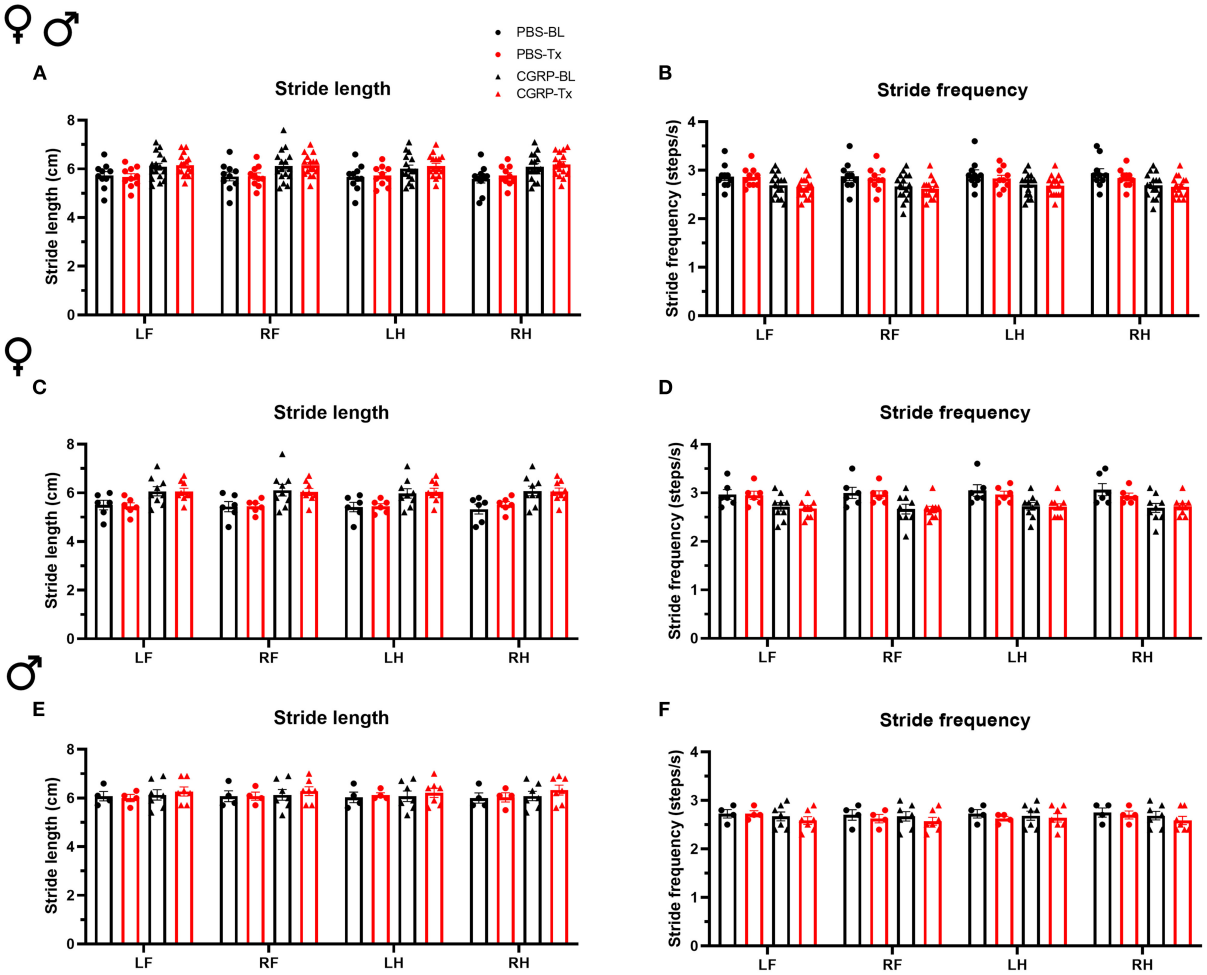
CGRP injection into the MN did not induce gait alterations. Stride length **(A)** and frequency **(B)** for all mice following injection of PBS (*n* = 10) or CGRP (1 μg/200 nl; *n* = 16) into the right MN of C57BL/6J mice via cannulas. Stride length **(C)** and frequency **(D)** for female mice (PBS: *n* = 6; CGRP: *n* = 9). Stride length **(E)** and frequency **(F)** for male mice (PBS: *n* = 4; CGRP: *n* = 7). Injection of CGRP into the MN did not change the stride length and frequency comparing before and after CGRP or PBS treatments across and within sexes. It suggests that CGRP in the MN did not change the gait. LF, left front paw; RF, right front paw; LH, left hind paw; RH, right hind paw. Data are the mean ± SEM. Statistics are described in Supplementary Table 1. Data are from one experiment.

Since the cerebellum is well-known for motor control and the MN controls axial and trunk muscles and maintains posture and balance (39), we tested the effect of CGRP delivery into the MN on gait. We conducted the gait dynamic assay using DigiGait system. Injection of PBS or CGRP into the MN did not change the stride length or frequency compared to their respective baselines across and within sexes (Figure 3). This indicates that CGRP administration into the MN decreases motility without gait alterations.

### Injection of CGRP Into the MN Induced Anxiety-Like Behavior in the Open Field Assay

To assess anxiety behavior independent of light, we used the open field assay. Inclusion of this assay was necessary because spending less time in the light in the light/dark assay can be an indicator of an increased anxiety state (44), and not necessarily a specific aversion to light. It is important to note though that the two are not mutually exclusive since light aversion may include increased anxiety.

**Figure 4:**
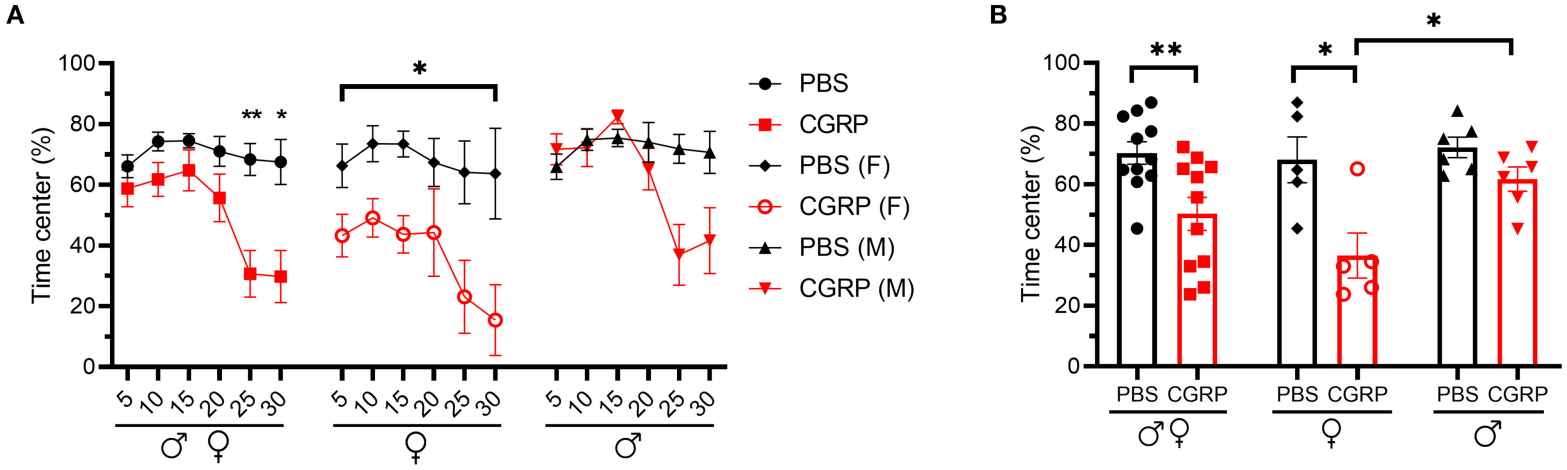
Injection of CGRP into the MN induced anxiety-like behavior in the open field assay. **(A)** Percentage of time spent in the center of the open field every 5-min block during 30-min testing period following injection of PBS (*n* = 11; F: *n* = 5; M: *n* = 6) or CGRP (1 μg/200 nl; *n* = 11; F: *n* = 5; M: *n* = 6) into the right MN of C57BL/6J mice via cannulas. All mice (left) separated by sex (female: middle; male: right). Data are from two independent experiments. All mice in A are further analyzed in B. **(B)** Mean percentage of time in the center per 5-min block for individual mice. Data are the mean ± SEM. Statistics are described in Supplementary Table 1. *p ≤ 0.05, **p ≤ 0.01.

The mice injected with CGRP spent less time in the center than those injected with PBS during the 30-min testing time (Figures 4A,B, left). However, when data were analyzed by sex, females exhibited significantly less time in the center over the entire testing time (Figures 4A,B, middle), while there was only a trend in males observed in the last 10 min (Figures 4A,B, right). Time in center of CGRP-treated females was significantly lower than CGRP-treated males (Figure 4B), which could contribute to the light-aversive behavior of the female mice.

### Injection of CGRP Into the MN Induced Plantar Tactile Hypersensitivity in the Contralateral Hind Paw

Cutaneous allodynia is present in ~60% of migraine patients with a higher prevalence in women than men (49). Thus, we investigated the effect of CGRP administration into the right MN on tactile hypersensitivity as a generally accepted indicator of allodynia by measuring the tactile sensitivity in the plantar area of the right and left hind paws.

**Figure 5:**
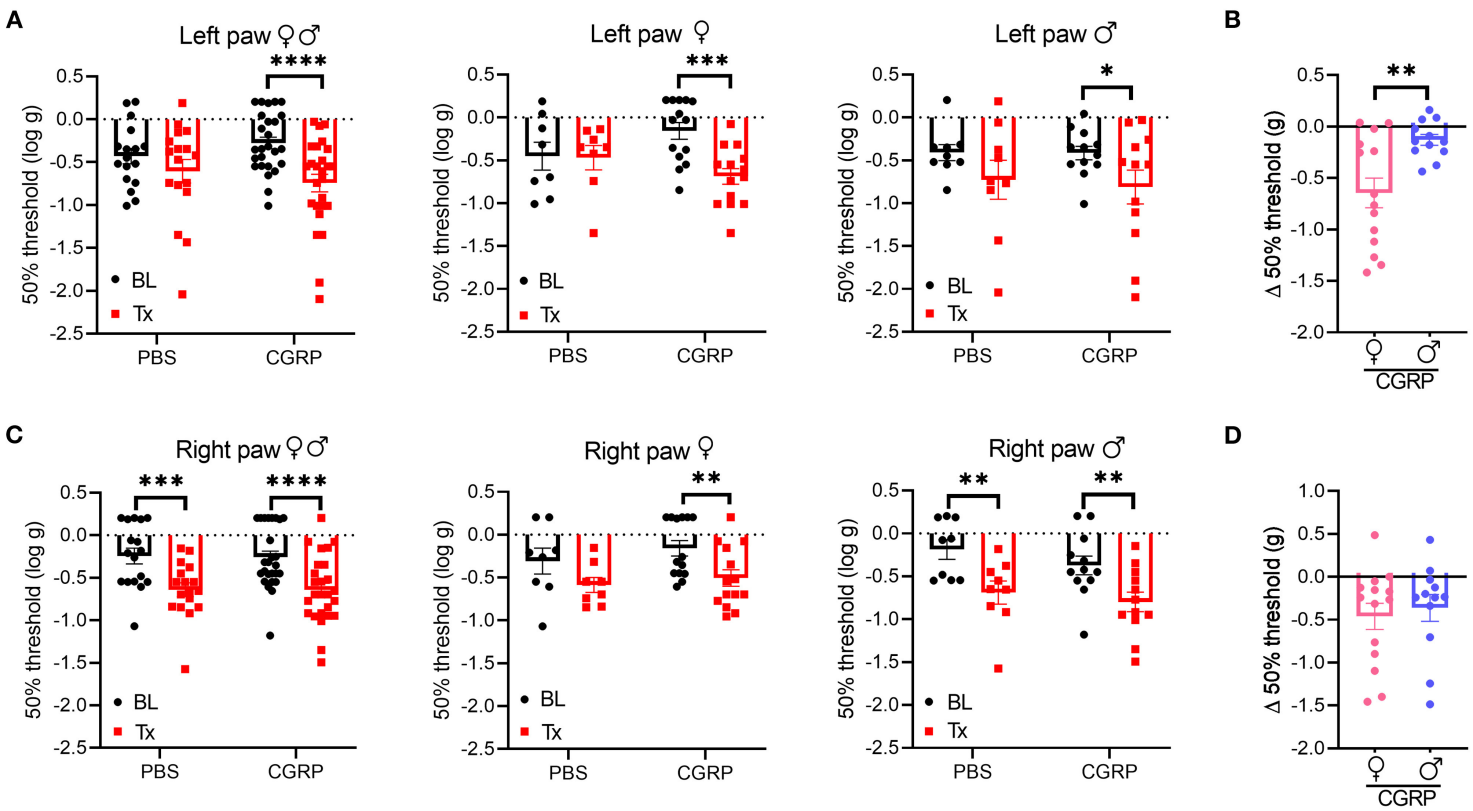
Injection of CGRP into the MN induced plantar tactile hypersensitivity in the contralateral hind paw. Plantar tactile sensitivity was assessed with injection of PBS or CGRP (1 μg/200 nl) into the right MN of C57BL/6J mice via cannulas. Data are from three independent experiments. **(A)** The individual thresholds of left hind paws for all mice (left) (PBS: *n* = 17; CGRP: *n* = 26), female mice (middle) (PBS: *n* = 8; CGRP: *n* = 14), and male mice (right) (PBS: *n* = 9; CGRP: *n* = 12). **(B)** Comparison of changes in withdrawal thresholds of left hind paws between CGRP-treated female and CGRP-treated male mice. The change in thresholds was measured by subtracting respective baseline from CGRP treatment measurements. **(C)** The individual thresholds of right hind paws for all mice (left) (PBS: *n* = 17; CGRP: *n* = 26), female mice (middle) (PBS: *n* = 8; CGRP: *n* = 14), and male mice (right) (PBS: *n* = 9; CGRP: *n* = 12). **(D)** Comparison of changes in withdrawal thresholds of right hind paws between CGRP-treated female and CGRP-treated male mice. The mean ± SEM 50% thresholds are presented. Statistics are described in Supplementary Table 1. *p ≤ 0.05, **p ≤ 0.01, ***p ≤ 0.001, ****p ≤ 0.0001.

In the contralateral left hind paw, there was a significant decrease in the withdrawal threshold observed for all the CGRP-treated mice (Figure 5A, left). When data were separated by sex, both female and male mice showed a significant decrease in the withdrawal threshold after CGRP but not PBS injection compared to their respective baselines (Figure 5A, middle and right). The sex difference was evaluated by comparing the change in withdrawal thresholds from baseline to CGRP injection. The threshold change in the female CGRP group was significantly larger that in male CGRP group (Figure 5B).

In contrast, the ipsilateral right paw results were more complicated due to a significant decrease in withdrawal threshold compared to baselines in response to not only CGRP, but also PBS vehicle (Figure 5C, left). When separated by sex, there was a trend for female mice after PBS treatment and a significant decrease after CGRP treatment compared to respective baselines (Figure 5C, middle). A significant decrease was observed for male mice after either PBS or CGRP treatment (Figure 5C, right) compared to baselines. The decrease in males after CGRP treatment is similar to the vehicle effect observed with PBS injection, suggesting that disturbance to the right MN is enough to increase ipsilateral hind paw sensitivity. No sex difference was observed in the threshold change in CGRP groups (Figure 5D). For the von Frey test, 7 of the 43 mice had injection sites not in or near the MN. When comparing data between on-target mice and off-target mice, no difference was observed between these two groups, but it should be noted that the off-target mice were underpowered. Altogether, these data suggest that CGRP increases the contralateral left hind paw touch sensitivity and this effect in females is more robust than males, while injection of either PBS vehicle or CGRP increases sensitivity in the ipsilateral right hind paw.

### Injection of CGRP Into the MN Induced Nociceptive Squinting Behavior

The grimace scale was developed to evaluate spontaneous pain expression in mice (50). Our laboratory found that orbital tightening, or squint, is the principal component of mouse grimace score (51) and has developed an automated video-based squint assay to measure spontaneous pain (48). Taking advantage of this sensitive automated squint platform, we asked whether mice squint after CGRP injection in the MN.

**Figure 6:**
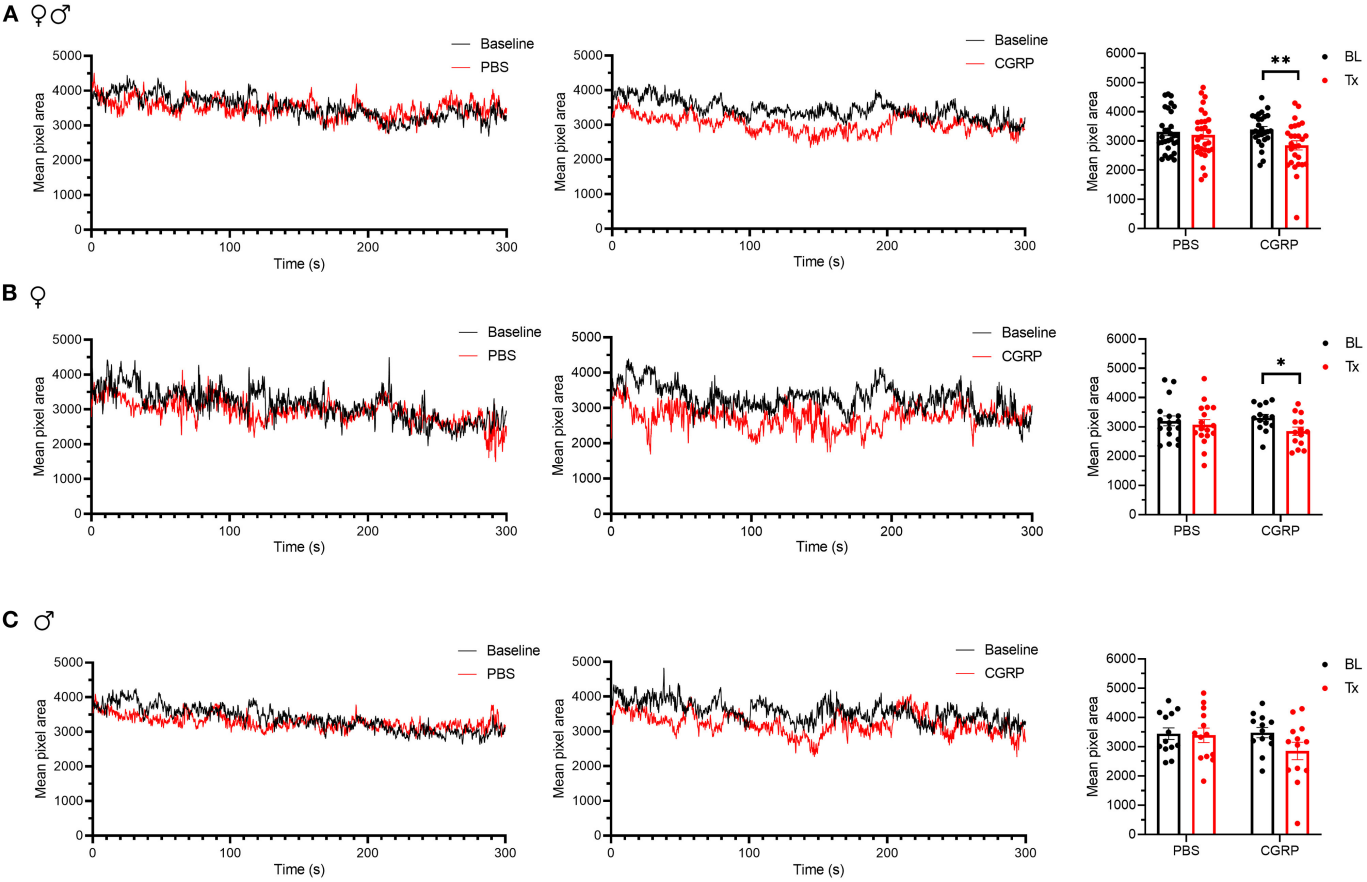
Injection of CGRP into the MN induced nociceptive squinting behavior. **(A)** Mean pixel area over 5-min testing period for all mice without treatment (as baseline), with injection of PBS (left) or CGRP (middle; 1 μg/200 nl) into the right MN of C57BL/6J mice via cannulas. Right panel is the mean pixel area over 5-min testing period for individual mice (PBS: *n* = 30; CGRP: *n* = 27). Data from A separated as female (B) and male (C). Data are from two independent experiments and one crossover treatment experiment. **(B)** Mean pixel area over 5-min testing period for female mice (left and middle) and mean pixel area over 5-min testing period for individual female mice (right; PBS: *n* = 17; CGRP: *n* = 14). **(C)** Mean pixel area over 5-min testing period for male mice (left and middle) and mean pixel area over 5-min testing period for individual male mice (right; PBS: *n* = 13; CGRP: *n* = 13). Data are the mean ± SEM. Statistics are described in Supplementary Table 1. *p ≤ 0.05, **p ≤ 0.01.

For all mice, CGRP-treated mice showed a decrease in the mean pixel area over the 300-s testing period, while no change was observed in the PBS-treated mice compared to their respective baselines (Figure 6A). When data were separated by sex, no difference was observed in female or male PBS-treated groups compared to respective baselines (Figures 6B,C, left and right). CGRP-treated females showed a significant decrease in the mean pixel area (Figure 6B, middle and right) while CGRP-treated males did not (Figure 6C, middle and right) compared to respective baselines. However, when male and female changes were directly compared in CGRP treatment groups, there was no significant difference (Supplementary Table 1). This may be attributed to males being underpowered based on *post-hoc* two-way repeated measure ANOVA power analysis. These data suggest that CGRP injection into the MN induces spontaneous pain in mice.

### The Diffusion Range of CGRP From the Injection Sites

**Figure 7:**
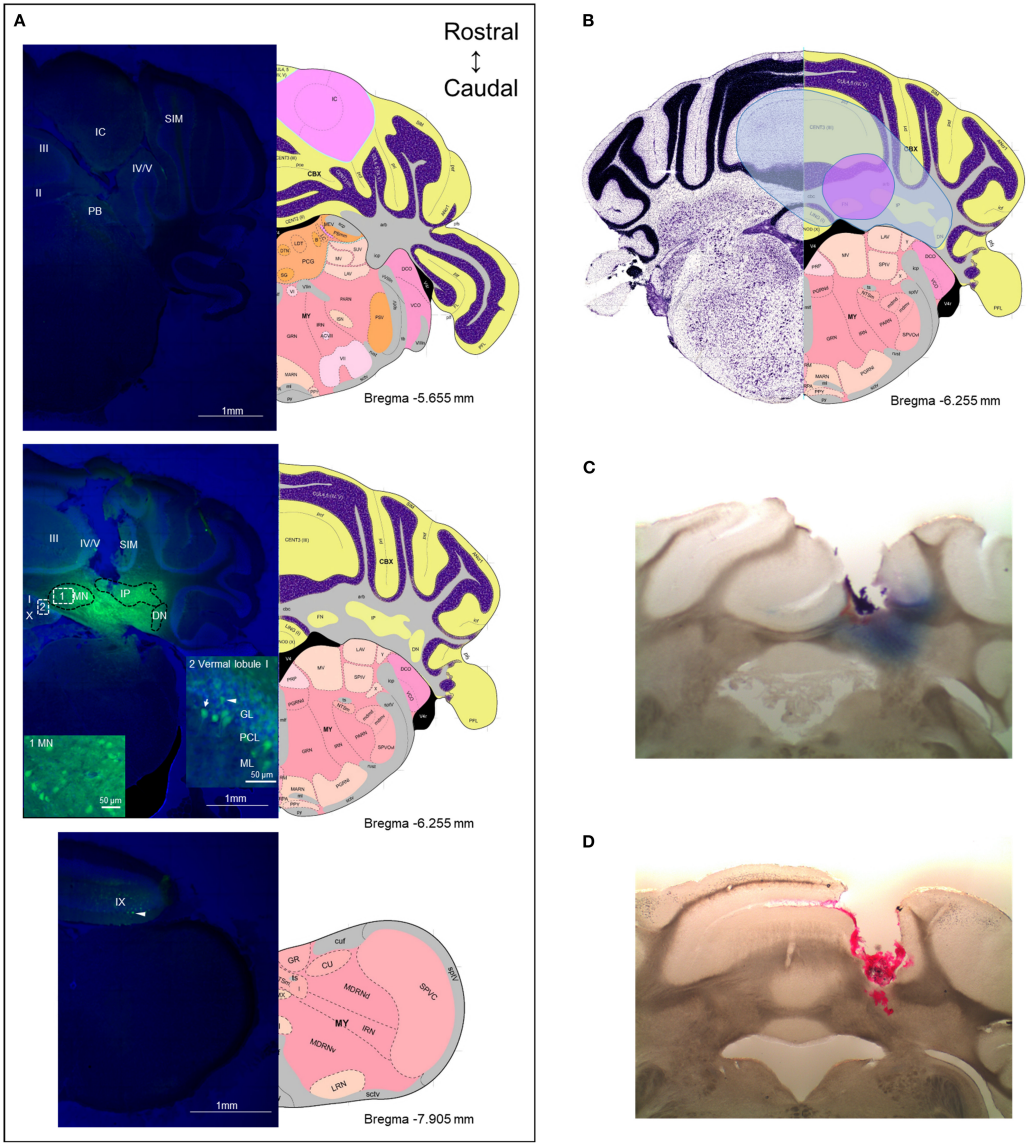
The diffusion range of CGRP. **(A)** Representative example of a mouse after injection of fluorescein-15-CGRP. Upper panel: In the most rostral section, dim fluorescence was detected in the inferior colliculus, the parabrachial nucleus, vermal lobules II-V, and the simple lobule. Middle panel: Fluorescein-15-CGRP at the injection site. Areas within rectangle are magnified in boxes 1 and 2. Clusters of fluorescein-15-CGRP were detected in cell bodies in the MN (box 1) and nearby cells, including the interposed and lateral cerebellar nuclei, granular, Purkinje cell, and molecular layers of vermal lobules I/III/IV/V (box 2). Dim signal was found in the simple lobule of the hemispheric regions. Lower panel: In the most caudal section, dim fluorescence was detected in lobule IX. Green: fluorescein-15-CGRP; Blue: TO-PRO-3. **(B)** The spread of the green fluorescence among the mice injected with fluorescein-15-CGRP. The smallest (purple shading) spread of signals covers the MN and few of nearby cells in the vermal lobules III/IV/V. The largest spread (blue shading) covers the MN and cells beyond the MN including vermal lobules I/III/IV/V/X, the simple lobule and other cerebellar deep nuclei. In summary, florescent signals were found in vermal lobules I-X, the simple lobule in the hemispheric region, and the midbrain (mainly in superior and inferior colliculus) from rostrally to caudally. **(C)** A representative image of a mouse with injection of Evans blue. **(D)** A representative image of a mouse with injection of red beads. DN, lateral cerebellar nucleus (dentate nucleus); GL, granular layer; IC, inferior colliculus; IP, interposed nucleus; ML, molecular layer; MN, medial cerebellar nucleus; PB, parabrachial nucleus; PCL, Purkinje cell layer; SIM, simple lobule. Image credit: Allen Institute. Numbers indicate the distance from bregma in the anteroposterior plane in Allen Mouse Brain Atlas coronal images.

To obtain an estimate of the likely diffusion of CGRP after injection into the MN, we used a fluorescent CGRP analog. Fluorescein-15-CGRP is a full CGRP receptor agonist but has less potency than CGRP as measured by cAMP production in HEK293T cells (52). Representative images of the rostral and caudal borders of fluorescein-15-CGRP diffusion from the MN are shown in Figure 7A upper and lower panels, respectively. There was considerable diffusion of fluorescein-15-CGRP from the injection site, with punctate signals found in cell bodies in the MN that may represent binding sites (Figure 7A, middle panel, box 1). In addition to the MN, fluorescein-15-CGRP was also observed in nearby regions, including the interposed and lateral cerebellar nuclei, granular, Purkinje cell, and molecular layers of vermal lobules I/III/IV/V (Figure 7A, middle panel, box 2). There was some variability in the spread of fluorescence among the mice injected with fluorescein-15-CGRP, with the smallest spread covering the MN and few nearby cells in the vermal lobules III/IV/V (Figure 7B, purple shading) and the largest spread covering the MN and cells beyond the MN including vermal lobules I/III/IV/V/X, the simple lobule and other cerebellar deep nuclei (Figure 7B, blue shading). The reason for variability in diffusion is not known but is apparently not due to injection site variability based on the injection sites shown by injection of red beads or Evans blue (Figures 7C,D).

## Discussion

To our knowledge, this is the first preclinical cerebellar study looking at behavioral outcomes related to migraine. Indeed, there have been few animal studies looking at imaging and electrophysiological links between the cerebellum and migraine (53–58). Brain imaging studies have reported that the cerebellar sodium concentration and functional connectivity to the insula or anterior cingulate cortex were altered in animal migraine models induced by nitroglycerin or inflammatory soup (53–55). The firing rate of Purkinje cells in the rat paraflocculus was decreased in an animal model induced by trigeminal stimulation (56), and the organization of parallel fibers to Purkinje cell synapses was abnormal in familial hemiplegic migraine type 1 mouse models (57, 58). There are also preclinical behavioral studies investigating the role of the cerebellum in pain modulation (41–43, 59–64). Our finding that several migraine-like symptoms can be induced by CGRP actions in the cerebellum supports the hypothesis that the cerebellum contributes to migraine pathogenesis.

### The MN and Light Aversion

Photophobia is a subjective experience in which normal light causes discomfort (65), and represents the most bothersome symptom in migraine patients other than pain (66). In this study, we found that administration of CGRP into the MN induced light aversion in male and female mice. In addition, CGRP evoked anxiety-like behavior in females, suggesting that in females the light aversion may be influenced by an overall increased anxiety-like state. In male mice, there appears to be a biphasic response where an anxiety-like response occurred during the final 10 min of the assay. While not significant, it suggests that the light aversion detected in males may be partially driven by increased anxiety at later time points. Moreover, there was a strong correlation between time in light and time in center (Supplementary Figure 2A), suggesting that light aversion is correlated to anxiety. This result is consistent with clinical studies that anxiety symptoms were positively correlated to light aversion in migraine patients with the possibility that anxiety contributes to light aversion (67).

An unexpected finding was that both male and female mice were aversive to even dim light after CGRP injection, analogous to migraine patients who report light hypersensitivity in dim light that does not bother control subjects. We had previously reported that transgenic mice overexpressing the CGRP receptor in the nervous system were sensitive to dim light (~55 lux) after i.c.v. CGRP injection (11, 13, 14), while light aversion induced in wild-type C57BL/6J mice required bright light (~27,000 lux, similar to a sunny day) (12). Those data suggested that hypersensitivity to CGRP in the nervous system leads to light hypersensitivity. Interestingly, in contrast to i.c.v. injections, CGRP injected directly into the posterior thalamic nuclei (15) and cerebellar MN in this study, caused light aversion with dim light in C57BL/6J mice. These data indicate that like the posterior thalamus, the MN is sensitive to CGRP signaling without a need to increase receptor expression, perhaps due to increased local concentrations of CGRP relative to i.c.v. deliveries.

One model to explain the clinical manifestation of photophobia is convergence of signals from intrinsically photosensitive retinal ganglion cells onto posterior thalamic neurons that also receive nociceptive signals from the trigeminal nucleus (68). Light and nociceptive signals are then integrated and sent to the somatosensory and visual cortices (68). In support of this model, we have recently reported that injection of CGRP into the posterior thalamic region or optogenetic stimulation of that same region caused light aversion (15). How might the cerebellum fit into this model? One possibility may be via bilateral fibers from the principal sensory trigeminal nucleus and spinal trigeminal nucleus to the posterior vermis of the cerebellum (32), which projects to the MN (39). The MN is known to project to various thalamic nuclei including parafascicular, centrolateral, mediodorsal, ventrolateral, suprageniculate, and posterior nuclei (40). Thus, the MN may lie in a circuit from the trigeminal system to the thalamus. However, unlike the thalamus, there are no apparent direct retinocerebellar connections (69, 70). These data place the MN in a prime position to assist in sensory integration and play a modulatory role in the nociceptive- and light- integrating function of the thalamus.

### The MN and Anxiety

We observed that anxiety-like behavior in response to cerebellar CGRP injection was only statistically significant in female mice. However, we want to couch that observation with the prediction that if more mice were analyzed, then the trends seen with male mice in the open field assay could possibly reach significance. Since this study was designed to be sufficiently powered (see Statistical Analysis), and the female group has a >80% power using a *post-hoc* power analysis, we did not try to further increase the number of male mice. To estimate how many more male mice might be required to reach significance, we did a *post-hoc* power analysis and found that about twice the number of male mice is predicted to be needed to reach a power of 80% comparable to females. Hence, as seen with human migraine populations, we are most likely seeing a quantitative bias for female responses and not an absolute female-only mechanism.

Like migraine, the apparent sexually dimorphic anxiety-like behaviors observed after CGRP injection into the MN are consistent with the higher prevalence of anxiety disorders in women than men (71). The MN sends direct projections to the limbic system including the amygdala (39), which is key to the anxiety circuitry (72), and projects to the periaqueductal gray (PAG) (40, 73), which is critical for aversive and anxiety-like responses (74). The observations of light-aversive behavior accompanied by increased anxiety levels in females are reminiscent of the behavior induced by optical stimulation of the dorsal PAG (15). This evidence might explain the anxiogenic effect of the MN. The possible mechanism for the sex difference in anxiety (or plantar tactile hypersensitivity discussed in The MN and Evoked and Spontaneous Pain) is not known. It is interesting to point out that sex differences appeared in human fMRI studies when migraine patients were exposed to a noxious stimulus (75). In that study, female migraine patients showed higher activation in the cerebellum and higher deactivation of cerebellar functional connectivity with insula than males in response to noxious heat. Finally, it is possible that there could be sexually dimorphic differences in the distribution pattern of CGRP receptor components in the MN or in downstream brain regions.

### The MN and Evoked and Spontaneous Pain

Allodynia is the perception of pain induced by non-noxious stimuli. Nearly 60% of individuals with migraine have cutaneous allodynia, specifically thermal and mechanical allodynia (49). Cutaneous allodynia is associated with migraine frequency, severity, and disability, and is more common in females (49). Moreover, cutaneous allodynia is more frequent in chronic migraine than episodic migraine (76) and is believed to be a predictor of migraine chronification (77). Allodynia in migraine is found in cephalic and extracephalic regions, which could be explained by the sensitization of the second-order trigeminal and third-order thalamic neurons (78).

In this study, we found that the increased sensitivity to mechanical stimuli in contralateral hind paws induced by CGRP in female mice was significantly greater than in male mice. This result is consistent with the clinical finding that cutaneous allodynia is higher in women than in men (49) and a preclinical study where intraplantar CGRP at a low dose evoked hind paw allodynia only in female rats (79). Given that we also observed anxiety behavior primarily in female mice, it is interesting that allodynia is associated with a higher risk for anxiety and a correlation exists between their severity in migraine patients (80). Anxiety was more prevalent in patients with migraine and probable migraine with cutaneous allodynia than those without cutaneous allodynia (81). In animal models, stress elicited higher pain sensitivity (82). These data suggest an association between anxiety and allodynia, so it is possible that anxiety induced by CGRP injection in female mice is linked to the tactile hypersensitivity indicative of allodynia. However, there was no apparent correlation between left paw withdrawal threshold changes and either time in center or time in light (Supplementary Figures 2B,C), although this conclusion must be tempered by the small number of paired mice.

How might the cerebellum increase paw sensitivity? There is evidence the cerebellum can affect the descending pain modulation pathway (59, 63, 64, 83), including via connections to the reticular formation (40, 59, 63). One study suggested that the MN could impact the dorsal column–medial lemniscus pathway directly or via the descending pain pathway (43). In addition, the MN projects to the thalamus bilaterally with contralateral preponderance (40), which might contribute to central sensitization and then lead to a pain hypersensitive state. However, the specific neuronal type in the MN that expresses CGRP receptors and specific regions that are modulated by CGRP in the MN are unknown.

An unexplained observation is that the ipsilateral hind paw showed a significant decrease in sensitivity after both PBS and CGRP injection into the MN. No change was observed after inserting the injection cannulas into the MN, without any injections, suggesting that the response was due to the solution. Because of the vehicle effect, a conclusion cannot be drawn from the ipsilateral paw data.

Our studies showed that CGRP injection into the MN induced nociceptive squinting behavior, suggesting CGRP in the MN plays a role in spontaneous pain. The magnitude of the squint response is relatively small (15%) compared to intraplantar injection of formalin (25%), and is closer to the response seen with wild-type female C57BL/6J mice receiving a small i.p. CGRP dose (0.01 mg/kg) (17%) (48). Similar to the open field assay, only female mice displayed a significant squint response after CGRP treatment. However, because there was no significant difference in the change in mean pixel area between CGRP-treated females and CGRP-treated males, we are hesitant to conclude that the squint response is female-specific. There was a correlation between time in light and changes in mean pixel area (Supplementary Figure 2D), suggesting a relationship between these two behaviors.

### The MN and Motor Function

We observed increased resting time in the dark while no change in the light in the light/dark assay across and within sex, corresponding to the preference of migraine patients to go to the dark and rest. Vertical beam breaks and transitions were decreased, suggesting exploratory behavior was decreased. The MN is responsible for controlling axial and trunk muscles, posture and balance (39). However, we did not observe gait difference before or after PBS or CGRP treatment using the DigiGait system. A recent study reported that an increase in light intensity could enhance postural sway in migraine patients compared to controls (29), so perhaps additional triggers may be needed to detect such effects in mouse models. Overall, these data suggest that CGRP injection into the MN does not induce gait changes.

### Caveats

A caveat of this study is the broad diffusion area of CGRP. We used fluorescein-15-CGRP for diffusion estimation. Extensive spread was observed from the MN with the injection of fluorescein-15-CGRP, which was not completely unexpected. The diffusion was similar (~800–4,000 μm rostral to caudal) as when fluorescein-15-CGRP was injected into the posterior thalamic region and was estimated to spread at least 1,400 μm in some cases (15). This is consistent with the volume transmission reported for some peptides diffusing up to millimeters in the brain (84). The robust spread of CGRP explains why even the off-target injections had similar behaviors as the on-target injections. While the diffusion is extensive, it apparently did not reach the fourth ventricle, which is near the MN, since no fluorescein-15-CGRP was detected in the fourth ventricle. Furthermore, the behavior is not likely due to CGRP diffusing into the cerebrospinal fluid since, as mentioned earlier, i.c.v. CGRP did not induce light aversion in wild-type C57BL/6J mice under dim light (12), while injection into the MN did. Nonetheless, the broad diffusion of fluorescein-15-CGRP beyond the MN decreases the targeting specificity, which makes it difficult to pinpoint which regions, in the MN or near the MN, are important for the responses induced by CGRP. For example, we noticed some dim fluorescence in the parabrachial nucleus (PB) (Figure 7A, upper panel). The PB, including CGRP-expressing neurons, are implicated in pain responses (85). While the florescence in the PB does not appear to be bright clusters indicative of CGRP binding or uptake, we cannot exclude the possibility that CGRP diffusion into the PB or other regions contributes to behaviors observed in this study. Future studies will need to focus on limiting the spread of CGRP beyond the MN.

A related caveat is that location of CGRP receptor subunits RAMP1 and CLR in the mouse cerebellum has not been studied. Importantly, clusters of fluorescein-15-CGRP observed within the MN are consistent with a prior report of MN CGRP receptors in the rat (37). Such clusters were also found in the molecular, Purkinje cell and granular layers in the vermal lobules and simple lobule in the hemisphere regions. Previous studies have reported RAMP1 and CLR co-expression in Purkinje cells in the rat, human and rhesus cerebellum (36, 38). However, consistent data is lacking for RAMP1 or/and CLR expression in the molecular layer or granular layer in the rat cerebellum (36, 37) and no reports for mice, to our knowledge. In addition, the possible expression of the second CGRP receptor (AMY1) (86) in the mouse cerebellum has not been explored.

While the use of an in-subject design allowed repeated delivery of CGRP or vehicle to the same mice and hence comparisons within the same mice, there were also drawbacks to this design. For example, we cannot rule out effects of repeated CGRP injections. To partly address this concern, we switched the order of light/dark and open field assays in two independent cohorts. The results were similar (data not shown). In addition, the order of experimentation was chosen to reduce effects on behavior. In our experience, the light aversion and open field assays are most prone to habituation, thus those assays were always done first. The automated squint assay is a stressful assay due to the use of a restraint, so it was always done last. Finally, we used a within-subject design for the plantar von Frey, gait dynamic and automated squint assays that allowed comparisons to baseline for each mouse. However, despite these precautions, we cannot exclude the possibility of cerebellar sensitization by multiple injections of CGRP.

A final caveat is that we will never know whether a mouse has a migraine. We are careful to call these phenotypes migraine-like. The key behavioral outputs in mouse migraine models are sensory phenotypes that are surrogates for photophobia, tactile hypersensitivity, and pain. Photophobia is the most bothersome symptom other than pain in migraine patients (66). Cephalic tactile sensitivity is the most common form of allodynia in patients with migraine. However, approximately 25–50% of individuals with migraine report extracephalic allodynia (78, 87–89). Among these studies, one study reported ~25% of patients had the extracephalic allodynia, either together with cephalic or on its own (mostly in the limbs) (87), suggestive of a significant proportion of patients suffering these symptoms. We attempted to measure periorbital sensitivity but have been unable to reproducibly habituate C57BL/6J mice to von Frey filaments in our laboratory. Therefore, we were limited to testing extracephalic (paw) sensitivity. Beyond behavior, future studies might examine trigeminal nucleus caudalis (TNC) activation after cerebellar CGRP injection. However, while the spinal trigeminal nuclei projects to the cerebellum (32), there apparently is not a direct reciprocal connection. Furthermore, peripheral CGRP injection did not increase TNC c-fos levels (90), although the same group found that a CGRP receptor antagonist blocked increased TNC c-fos in glyceryl trinitrate-treated rats (91). Given the complexity of data regarding cerebellar-TNC connections and peripheral CGRP actions on the TNC, further studies are needed to unravel how the cerebellum might communicate with the trigeminal system in migraine.

## Conclusions

In conclusion, this study reveals that CGRP injection into the cerebellum is sufficient to induce migraine-like behaviors in mice, although the effects on anxiety and tactile hypersensitivity are more prominent in females. This discovery provides a new perspective on the increasingly complex neural circuitry of migraine and further implicates central actions of CGRP in migraine pathophysiology.

## Data Availability Statement

The raw data supporting the conclusions of this article will be made available by the authors, without undue reservation.

## Ethics Statement

The animal study was reviewed and approved by University of Iowa IACUC and Iowa City Veteran Health Care System IACUC.

## Author Contributions

MW, LS, and AR designed research. MW, TD, BR, JW, MH, and HF performed research. MW, LS, and TD analyzed data. MW, LS, TD, and AR interpreted data. MW, LS, and AR wrote the paper. All authors contributed to the article and approved the submitted version.

## Funding

This work was supported by grants from the NIH R01 NS075599 and RF1 NS113839, VA-ORD (RR&D) MERIT 1 I01 RX003523-0, Career Development Award (IK2 RX002010), and Center for Prevention and Treatment of Visual Loss (VA C6810-C).

## Author Disclaimer

The contents do not represent the views of Veterans Administration or the United States Government.

## Conflict of Interest

AR is a consultant for Lundbeck, Amgen, Novartis, Eli Lilly, AbbVie, and Schedule 1 Therapeutics. The remaining authors declare that the research was conducted in the absence of any commercial or financial relationships that could be construed as a potential conflict of interest.

## Publisher's Note

All claims expressed in this article are solely those of the authors and do not necessarily represent those of their affiliated organizations, or those of the publisher, the editors and the reviewers. Any product that may be evaluated in this article, or claim that may be made by its manufacturer, is not guaranteed or endorsed by the publisher.
