# Supplementary material for: CGRP Administration Into the Cerebellum Evokes Light Aversion, Tactile Hypersensitivity, and Nociceptive Squint in Mice
Source: Front Pain Res (Lausanne). 2022 Apr 25;3:861598. doi: 10.3389/fpain.2022.861598 (PMC9082264; doi:10.3389/fpain.2022.861598)
Supplement: Supplementary file 1 [file Data_Sheet_1.pdf]

Supplementary Material

1.1 Supplementary Figures

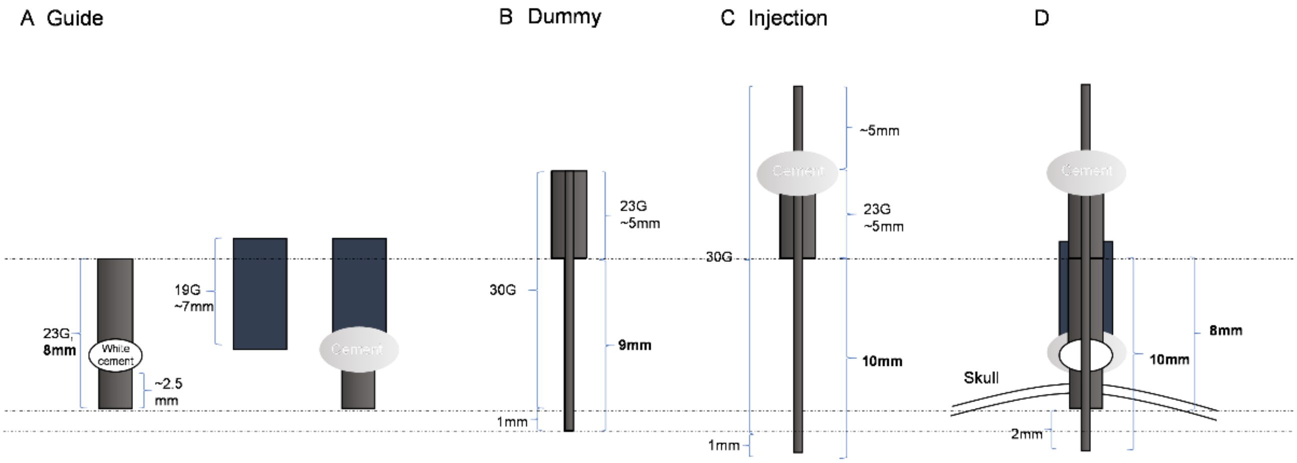

Supplementary Fig. 1 Cannula design and assembly diagram. (**A**) Guide cannulas. The guide cannula was made from an 8-mm, 23-gauge needle (left) with the ventral portion covered by a ~7-mm, 19-gauge tubing (middle), which were adhered by adhesive and dental cement (right). The plastic holder was removed from 23-gauge needle without removing the original white cement (left). The ~7-mm, 19-gauge tubing is ~2 mm higher than the 23-gauge needle to shield the junction between guide's top and the dummy or injection cannula after their insertion, allowing the dummy or the injection cannula to stay in position after insertion (right). (**B**) Dummy cannulas. The dummy cannula, used to seal and keep the guide cannula free of clogs, was made by crimping a short segment of ~5-mm, 23-gauge tubing to a ~14 mm piece of 30-gauge tubing. The bottom of the 30-gauge tubing was cut to ensure that the 30-gauge segment below the ~5-mm, 23-gauge segment is 9 mm. (**C**) Injection cannulas. The injection cannula was made by adhering a short segment of ~5-mm, 23-gauge tubing ~5 mm below the top of a ~20-mm piece of 30-gauge tubing with adhesive and dental cement. The bottom of the 30-gauge tubing was cut to ensure that the 30-gauge segment below the ~5-mm, 23-gauge segment is 10 mm. (**D**) The injection cannula extended 2 mm beyond the base of the guide cannula when was inserted into the guide cannula.

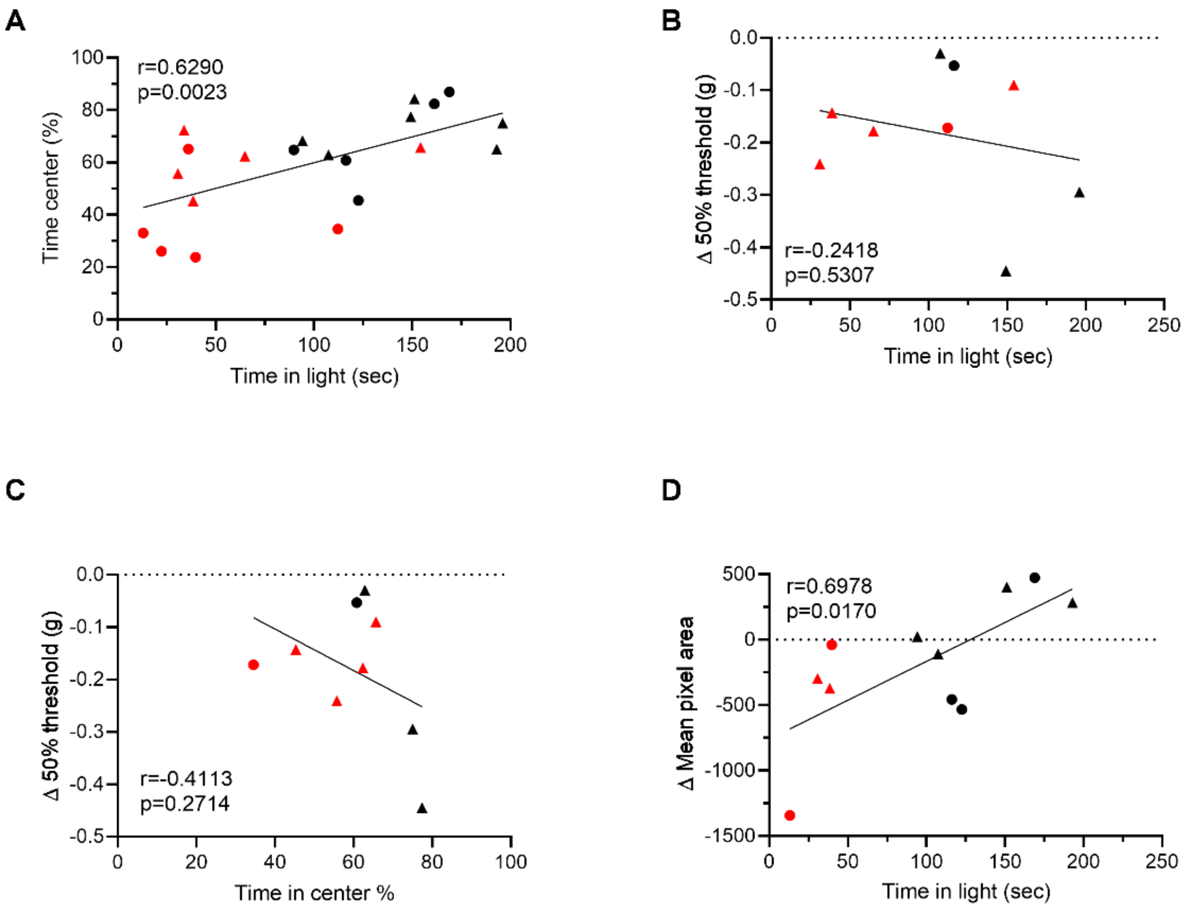

Supplementary Fig. 2 Correlation of behavioral outputs. (**A**) Correlation between time in light and time in center. PBS (n=11) or CGRP (n=10). (**B**) Correlation between time in light and changes in the left paw withdrawal threshold. PBS (n=4) or CGRP (n=5). (**C**) Correlation between time in center and changes in the left paw withdrawal threshold. PBS (n=4) or CGRP (n=5). (**D**) Correlation between time in light and changes in mean pixel area. PBS (n=7) or CGRP (n=4). Black circle (●) represents PBS-treated female mice; black triangle (▲) represents PBS-treated male mice; red circle (●) represents CGRP-treated female mice; red triangle (▲) represents CGRP-treated male mice. Statistics are described in Supplementary Table 1.

Supplementary Table 1 Statistical analyses

Figure no.	Analysis	Statistics (symbol on Figure)
Fig. 1A left (all mice)	Two-way repeated measure ANOVA	
	Interaction factor	F (5, 95) = 1.734, P=0.1343
	Treatment factor	F (1, 19) = 23.23, P=0.0001
	Time factor	F (2.677, 50.86) = 6.280, P=0.0015
Fig. 1A middle (females)	Two-way repeated measure ANOVA	
	Interaction factor	F (5, 40) = 0.7746, P=0.5738
	Treatment factor	F (1, 8) = 14.45, P=0.0052
	Time factor	F (2.675, 21.40) = 9.368, P=0.0005
Fig. 1A right (males)	Two-way repeated measure ANOVA	
	Interaction factor	F (5, 45) = 1.074, P=0.3877
	Treatment factor	F (1, 9) = 8.808, P=0.0158
	Time factor	F (2.076, 18.68) = 1.891, P=0.1777
Fig. 1B left (all mice)	Unpaired 2-tailed t-test	t=4.820, df=19, P=0.0001
Fig. 1B middle (females)	Unpaired 2-tailed t-test	t=3.802, df=8, P=0.0052
Fig. 1B right (males)	Unpaired 2-tailed t-test	t=2.968, df=9, P=0.0158
Fig. 1B<br>Female CGRP vs.<br>Male CGRP	Unpaired 2-tailed t-test	t=0.6791, df=8, P=0.5163
Fig. 2A upper panel		
Left (all mice)	Two-way repeated measure ANOVA (mixed effects analysis)	
	Interaction factor	F (5, 84) = 0.7322, P=0.6013
	Treatment factor	F (1, 19) = 0.01297, P=0.9105
	Time factor	F (2.807, 47.16) = 8.859, P=0.0001
Middle (females)	Two-way repeated measure ANOVA (mixed effects analysis)	
	Interaction factor	F (5, 36) = 0.4571, P=0.8053
	Treatment factor	F (1, 8) = 0.6466, P=0.4446
	Time factor	F (1.952, 14.05) = 7.334, P=0.0068
Right (males)	Two-way repeated measure ANOVA (mixed effects analysis)	
	Interaction factor	F (5, 38) = 1.570, P=0.1918
	Treatment factor	F (1, 9) = 0.7938, P=0.3961
	Time factor	F (1.714, 13.03) = 6.755, P=0.0119
Fig. 2A lower panel		
Left (all mice)	Two-way repeated measure ANOVA	
	Interaction factor	F (5, 95) = 2.995, P=0.0148
	Treatment factor	F (1, 19) = 28.00, P<0.0001
	Time factor	F (3.220, 61.18) = 41.21, P<0.0001
	Šídák's multiple comparisons test	*P < .05, **P < .01, ***P < .001
Middle (females)	Two-way repeated measure ANOVA	
	Interaction factor	F (5, 40) = 4.713, P=0.0018
	Treatment factor	F (1, 8) = 17.02, P=0.0033
	Time factor	F (2.779, 22.23) = 31.66, P<0.0001
	Šídák's multiple comparisons test	*P < .05, **P < .01
Right (males)	Two-way repeated measure ANOVA	
	Interaction factor	F (5, 45) = 0.6068, P=0.6950
	Treatment factor	F (1, 9) = 10.24, P=0.0108
	Time factor	F (2.691, 24.22) = 15.43, P<0.0001
Fig. 2B upper panel		
Left (all mice)	Unpaired 2-tailed t-test	t=0.06504, df=19, P=0.9488
Middle (females)	Unpaired 2-tailed t-test	t=0.9003, df=8, P=0.3942
Right (males)	Unpaired 2-tailed t-test	t=0.7370, df=9, P=0.4799
Fig. 2B lower panel		
Left (all mice)	Unpaired 2-tailed t-test	t=5.291, df=19, P<0.0001
Middle (females)	Unpaired 2-tailed t-test	t=4.125, df=8, P=0.0033
Right (males)	Unpaired 2-tailed t-test	t=3.200, df=9, P=0.0108

Fig. 2C upper panel					
Left (all mice)	Two-way repeated measure ANOVA (mixed effects analysis)				
	Interaction factor	F (5, 84) = 1.411, P=0.2288			
	Treatment factor	F (1, 19) = 4.694, P=0.0432			
	Time factor	F (2.759, 46.35) = 0.5223, P=0.6542			
Middle (females)	Two-way repeated measure ANOVA (mixed effects analysis)				
	Interaction factor	F (5, 36) = 2.016, P=0.0997			
	Treatment factor	F (1, 8) = 3.530, P=0.0971			
	Time factor	F (1.482, 10.67) = 0.1453, P=0.8048			
Right (males)	Two-way repeated measure ANOVA (mixed effects analysis)				
	Interaction factor	F (5, 38) = 0.9688, P=0.4491			
	Treatment factor	F (1, 9) = 1.575, P=0.2411			
	Time factor	F (1.905, 14.48) = 0.9849, P=0.3933			
Fig. 2C lower panel					
Left (all mice)	Two-way repeated measure ANOVA				
	Interaction factor	F (5, 95) = 1.020, P=0.4104			
	Treatment factor	F (1, 19) = 4.554, P=0.0461			
	Time factor	F (1.690, 32.10) = 5.394, P=0.0129			
Middle (females)	Two-way repeated measure ANOVA				
	Interaction factor	F (5, 40) = 2.197, P=0.0737			
	Treatment factor	F (1, 8) = 3.328, P=0.1055			
	Time factor	F (1.459, 11.67) = 4.477, P=0.0453			
Right (males)	Two-way repeated measure ANOVA				
	Interaction factor	F (5, 45) = 0.4386, P=0.8192			
	Treatment factor	F (1, 9) = 1.189, P=0.3039			
	Time factor	F (2.333, 20.99) = 2.168, P=0.1332			
Fig. 2D upper panel					
Left (all mice)	Unpaired 2-tailed t-test	t=2.300, df=19, P=0.0330			
Middle (females)	Unpaired 2-tailed t-test	t=1.979, df=8, P=0.0831			
Right (males)	Unpaired 2-tailed t-test	t=1.321, df=9, P=0.2192			
Fig. 2D lower panel					
Left (all mice)	Unpaired 2-tailed t-test	t=2.131, df=19, P=0.0464			
Middle (females)	Unpaired 2-tailed t-test	t=1.854, df=8, P=0.1008			
Right (males)	Unpaired 2-tailed t-test	t=1.084, df=9, P=0.3066			
Fig. 2E					
Left (all mice)	Two-way repeated measure ANOVA				
	Interaction factor	F (5, 95) = 2.190, P=0.0617			
	Treatment factor	F (1, 19) = 19.19, P=0.0003			
	Time factor	F (3.507, 66.63) = 7.639, P<0.0001			
Middle (females)	Two-way repeated measure ANOVA				
	Interaction factor	F (5, 40) = 2.181, P=0.0755			
	Treatment factor	F (1, 8) = 10.05, P=0.0132			
	Time factor	F (2.947, 23.58) = 4.349, P=0.0145			
Right (males)	Two-way repeated measure ANOVA				
	Interaction factor	F (5, 45) = 0.6413, P=0.6694			
	Treatment factor	F (1, 9) = 8.275, P=0.0183			
	Time factor	F (2.478, 22.31) = 3.553, P=0.0375			
Fig. 2F					
Left (all mice)	Unpaired 2-tailed t-test	t=4.380, df=19, P=0.0003			
Middle (females)	Unpaired 2-tailed t-test	t=3.170, df=8, P=0.0132			
Right (males)	Unpaired 2-tailed t-test	t=2.877, df=9, P=0.0183			
		LF	RF	LH	RH
Fig. 3A (all mice)	Two-way repeated measure ANOVA				
	Interaction factor	F (1, 24) = 0.6154, P=0.4404	F (1, 24) = 0.01717, P=0.8968	F (1, 24) = 0.08938, P=0.7675	F (1, 24) = 0.02838, P=0.8676
	Treatment factor	F (1, 24) = 5.359, P=0.0295	F (1, 24) = 4.852, P=0.0375	F (1, 24) = 4.018, P=0.0564	F (1, 24) = 6.202, P=0.0201
	Condition factor	F (1, 24) = 0.0006404, P=0.9800	F (1, 24) = 0.06471, P=0.8014	F (1, 24) = 1.430, P=0.2434	F (1, 24) = 1.882, P=0.1828
Fig. 3B (all mice)	Two-way repeated measure ANOVA				

	Interaction factor	F (1, 24) = 0.5979, P=0.4469	F (1, 24) = 2.546e-029, P>0.9999	F (1, 24) = 1.158, P=0.2926	F (1, 24) = 1.026, P=0.3213
	Treatment factor	F (1, 26) = 5.467, P=0.0280	F (1, 24) = 5.044, P=0.0342	F (1, 24) = 4.075, P=0.0548	F (1, 24) = 5.840, P=0.0236
	Condition factor	F (1, 24) = 1.227, P=0.2790	F (1, 24) = 1.748, P=0.1986	F (1, 24) = 3.624, P=0.0690	F (1, 24) = 2.849, P=0.1044
Fig. 3C (females)	Two-way repeated measure ANOVA				
	Interaction factor	F (1, 13) = 0.04835, P=0.8294	F (1, 13) = 0.1587, P=0.6969	F (1, 13) = 0.04021, P=0.8442	F (1, 13) = 1.409, P=0.2565
	Treatment factor	F (1, 13) = 6.555, P=0.0237	F (1, 13) = 6.113, P=0.0280	F (1, 13) = 6.307, P=0.0260	F (1, 13) = 7.275, P=0.0183
	Condition factor	F (1, 13) = 0.1194, P=0.7352	F (1, 13) = 0.05712, P=0.8148	F (1, 13) = 0.3619, P=0.5578	F (1, 13) = 0.8653, P=0.3692
Fig. 3D (females)	Two-way repeated measure ANOVA				
	Interaction factor	F (1, 13) = 0.04216, P=0.8405	F (1, 13) = 0.05000, P=0.8265	F (1, 13) = 0.7695, P=0.3963	F (1, 13) = 3.145, P=0.0996
	Treatment factor	F (1, 13) = 5.438, P=0.0364	F (1, 13) = 7.020, P=0.0200	F (1, 13) = 7.021, P=0.0200	F (1, 13) = 6.418, P=0.0250
	Condition factor	F (1, 13) = 0.3795, P=0.5485	F (1, 13) = 0.2000, P=0.6621	F (1, 13) = 1.316, P=0.2720	F (1, 13) = 1.132, P=0.3067
Fig. 3E (males)	Two-way repeated measure ANOVA				
	Interaction factor	F (1, 9) = 0.6454, P=0.4425	F (1, 9) = 0.3455, P=0.5711	F (1, 9) = 0.03195, P=0.8621	F (1, 9) = 0.5894, P=0.4623
	Treatment factor	F (1, 9) = 0.3741, P=0.5559	F (1, 9) = 0.2286, P=0.6439	F (1, 9) = 0.06064, P=0.8110	F (1, 9) = 0.5216, P=0.4885
	Condition factor	F (1, 9) = 0.06262, P=0.8080	F (1, 9) = 0.3455, P=0.5711	F (1, 9) = 1.026, P=0.3376	F (1, 9) = 0.8910, P=0.3698
Fig. 3F (males)	Two-way repeated measure ANOVA				
	Interaction factor	F (1, 9) = 0.8070, P=0.3924	F (1, 9) = 0.04120, P=0.8437	F (1, 9) = 0.3445, P=0.5717	F (1, 9) = 0.1848, P=0.6774
	Treatment factor	F (1, 9) = 0.6519, P=0.4403	F (1, 9) = 0.1070, P=0.7511	F (1, 9) = 0.007599, P=0.9324	F (1, 9) = 0.5434, P=0.4798
	Condition factor	F (1, 9) = 0.8070, P=0.3924	F (1, 9) = 2.019, P=0.1891	F (1, 9) = 2.153, P=0.1763	F (1, 9) = 1.663, P=0.2294
Fig. 4A left (all mice)	Two-way repeated measure ANOVA				
	Interaction factor	F (5, 100) = 5.414, P=0.0002			
	Treatment factor	F (1, 20) = 9.155, P=0.0067			
	Time factor	F (3.129, 62.59) = 9.223, P<0.0001			
	Šídák's multiple comparisons test	*P < .05, **P < .01			
Fig. 4A middle (females)	Two-way repeated measure ANOVA				
	Interaction factor	F (5, 40) = 1.270, P=0.2960			
	Treatment factor	F (1, 8) = 8.899, P=0.0175			
	Time factor	F (3.397, 27.17) = 3.397, P=0.0276			
Fig. 4A right (males)	Two-way repeated measure ANOVA				
	Interaction factor	F (5, 50) = 4.846, P=0.0011			
	Treatment factor	F (1, 10) = 4.013, P=0.0730			
	Time factor	F (2.374, 23.74) = 5.795, P=0.0065			
Fig. 4B left (all mice)	Unpaired 2-tailed t-test	t=3.026, df=20, P=0.0067			
Fig. 4B middle (females)	Unpaired 2-tailed t-test	t=2.983, df=8, P=0.0175			
Fig. 4B right (males)	Unpaired 2-tailed t-test	t=2.003, df=10, P=0.0730			
Fig. 4B Female CGRP vs. Male CGRP	Unpaired 2-tailed t-test	t=3.133, df=9, P=0.0121			
Fig. 5A left (all mice)	Two-way repeated measure ANOVA				
	Interaction factor	F (1, 41) = 3.684, P=0.0619			
	Treatment factor	F (1, 41) = 0.003699, P=0.9518			
	Condition factor	F (1, 41) = 17.87, P=0.0001			
	Paired 2-tailed t-test	****P < 0.0001			
Fig. 5A middle (females)	Two-way repeated measure ANOVA				
	Interaction factor	F (1, 20) = 8.429, P=0.0088			
	Treatment factor	F (1, 20) = 0.06922, P=0.7952			
	Condition factor	F (1, 20) = 9.630, P=0.0056			
	Paired 2-tailed t-test	***P < .001			
Fig. 5A right (males)	Two-way repeated measure ANOVA				
	Interaction factor	F (1, 19) = 0.1018, P=0.7532			
	Treatment factor	F (1, 19) = 0.05351, P=0.8195			
	Condition factor	F (1, 19) = 8.113, P=0.0103			
	Paired 2-tailed t-test	*P < .05			
Fig. 5B Female CGRP vs. Male CGRP	Unpaired 2-tailed t-test	t=3.170, df=24, P=0.0041			
Fig. 5C left (all mice)	Two-way repeated measure ANOVA				

	Interaction factor	F (1, 41) = 0.01288, P=0.9102
	Treatment factor	F (1, 41) = 0.005324, P=0.9422
	Condition factor	F (1, 41) = 44.42, P<0.0001
	Paired 2-tailed t-test	***P < .001, ****P < .0001
Fig. 5C middle (females)	Two-way repeated measure ANOVA	
	Interaction factor	F (1, 20) = 0.1767, P=0.6787
	Treatment factor	F (1, 20) = 0.7693, P=0.3908
	Condition factor	F (1, 20) = 14.41, P=0.0011
	Paired 2-tailed t-test	**P < .01
Fig. 5C right (males)	Two-way repeated measure ANOVA	
	Interaction factor	F (1, 19) = 0.2071, P=0.6542
	Treatment factor	F (1, 19) = 1.028, P=0.3233
	Condition factor	F (1, 19) = 30.49, P<0.0001
	Paired 2-tailed t-test	**P < .01
Fig. 5D<br>Female CGRP vs.<br>Male CGRP	Unpaired 2-tailed t-test	t=0.4501, df=24, P=0.6567
Fig. 6A right (all mice)	Two-way repeated measure ANOVA	
	Interaction factor	F (1, 55) = 4.902, P=0.0310
	Treatment factor	F (1, 55) = 0.7006, P=0.4062
	Condition factor	F (1, 55) = 10.26, P=0.0023
	Paired 2-tailed t-test	**P < .01
Fig. 6B right (females)	Two-way repeated measure ANOVA	
	Interaction factor	F (1, 29) = 2.756, P=0.1077
	Treatment factor	F (1, 29) = 0.08961, P=0.7668
	Condition factor	F (1, 29) = 9.036, P=0.0054
	Paired 2-tailed t-test	*P < .05
Fig. 6C right (males)	Two-way repeated measure ANOVA	
	Interaction factor	F (1, 24) = 2.309, P=0.1417
	Treatment factor	F (1, 24) = 0.8393, P=0.3687
	Condition factor	F (1, 24) = 3.282, P=0.0826
Changes in mean pixel area:<br>Female CGRP vs.<br>Male CGRP	Unpaired 2-tailed t-test	t=0.5501, df=25, P=0.5871
Suppl. Fig. 2A	Pearson correlation	r=0.6290, P=0.0023
Suppl. Fig. 2B	Pearson correlation	r=-0.2418, P=0.5307
Suppl. Fig. 2C	Pearson correlation	r=-0.4113, P=0.2714
Suppl. Fig. 2D	Pearson correlation	r=0.6978, P=0.0170
